# Particulate Matter-Induced Acute Coronary Syndrome: MicroRNAs as Microregulators for Inflammatory Factors

**DOI:** 10.1155/2021/6609143

**Published:** 2021-12-11

**Authors:** Nur Izah Ab Razak, Nor Eliani Ezani, Norzian Ismail

**Affiliations:** ^1^Department of Human Anatomy, Faculty of Medicine and Health Sciences, Universiti Putra Malaysia, 43400 Serdang, Malaysia; ^2^Department of Environmental and Occupational Health, Faculty of Medicine and Health Sciences, Universiti Putra Malaysia, 43400 Serdang, Malaysia; ^3^Department of Medicine, Faculty of Medicine and Health Sciences, Universiti Putra Malaysia, 43400 Serdang, Malaysia

## Abstract

The most prevalent cause of mortality and morbidity worldwide is acute coronary syndrome (ACS) and its consequences. Exposure to particulate matter (PM) from air pollution has been shown to impair both. Various plausible pathogenic mechanisms have been identified, including microRNAs (miRNAs), an epigenetic regulator for gene expression. Endogenous miRNAs, average 22-nucleotide RNAs (ribonucleic acid), regulate gene expression through mRNA cleavage or translation repression and can influence proinflammatory gene expression posttranscriptionally. However, little is known about miRNA responses to fine PM (PM_2.5_, PM_10_, ultrafine particles, black carbon, and polycyclic aromatic hydrocarbon) from air pollution and their potential contribution to cardiovascular consequences, including systemic inflammation regulation. For the past decades, microRNAs (miRNAs) have emerged as novel, prospective diagnostic and prognostic biomarkers in various illnesses, including ACS. We wanted to outline some of the most important studies in the field and address the possible utility of miRNAs in regulating particulate matter-induced ACS (PMIA) on inflammatory factors in this review.

## 1. Introduction

Air pollution has been considered a major public health threat and has caused about seven million deaths every year, according to the World Health Organization (WHO) [1]. The complex mixture of air pollutants arises from particulate matter (PM), chemical substances, and biological materials which are emitted from a natural process (volcano, oceans, forest, etc.) or anthropogenic activities (industry or transportation) [2]. With the increasing demands of global energy, the generation of the combustion of fossil fuels, including coal, diesel fuel, gasoline, and natural gas for electricity, heating, industry, and transportation, is also increased [3]. This has resulted in the higher release of air pollutants whereby air pollution was a major burden to an individual, especially to those living in the growing economic countries [4]. Of these, it is greatly important to address air pollution issues where reducing air pollution will meet the Sustainable Development Goals (SDG) proposed by the United Nation (UN) in SDG 3, for good health and well-being [5]. Increased PM from air pollution contributes to the occurrence of acute coronary syndrome (ACS) in several studies [6]. MicroRNAs (miRNAs), a short noncoding RNA, have been observed in few studies to be dysregulated in inflammatory modulation in cardiovascular disorder exposed to PM [7–9]. The aim of this review is to look at the potential role of miRNAs in the regulation of inflammatory factors in particulate matter-induced ACS (PMIA).

The references of this narrative review paper were searched through PubMed and Google Scholar. The keywords used were particulate matter, air pollution, acute coronary syndrome, miRNA, and inflammation.

### 1.1. Common Air Pollutants

There is growing awareness of health outcomes related to air pollution from short- and long-term exposure to air pollutants. Many studies have reported the adverse health effects linked with heart diseases from these exposures [10–13]. This showed how crucial the air pollutants' role is in cardiovascular events and outcomes. PM can be differentiated by ranging size of aerodynamic diameter (AD) from 2.5 to 10 *μ*m (including PM_2.5_ and PM_10_) and chemical composition (including black carbon, polycyclic aromatic hydrocarbon, and trace metals). Organic aerosol is mainly the result of anthropogenic emissions such as vehicle emissions, residential fuel combustion, and wildfires, representing a large proportion of PM. It is estimated that 180,000 (117,000 to 389,000) premature deaths were prevented from reducing anthropogenic organic aerosol between 1990 and 2012 [14].  Table 1 shows the summary of the sources of PM_2.5_, PM_10_, ultrafine particle (UFP, AD less than 0.1 *μ*m), black carbon (BC), and polycyclic aromatic hydrocarbons (PAHs). According to a study conducted in a medium-sized Dutch city, UFP and BC concentrations in transportation zones more than doubled between 8 a.m. and 10 a.m. compared to those recorded at an urban background location [15]. In PM_2.5_ containing the ultrafine fraction, PAH concentrations were 12-fold higher than M_2.5-0.3_, which may be explained by combustion processes that produce ultrafine particles containing high concentrations of PAHs [16, 17]. Among the most significant ultrafine particle (UFP) sources in urbanised areas are diesel combustion and solid biomass combustion [18]. Biomarkers associated with inflammation (*CCXL2*, *EPGN*, *GREM1*, *IL-1α*, *IL-1β*, *IL-6*, *IL-24*, *EREG*, and *VEGF*) and transcription factors (NFE2L2, MAFF, HES1, FOSL1, and TGIF1) relevant for cardiovascular and lung disease are secreted in response to diesel UFP exposure [19]. According to one in vitro study, PM_2.5_ increased gene alteration, DNA damage, cytotoxicity, and reactive oxygen species (ROS) in the A549 cell line, most likely due to CYP enzyme activation in response to polycyclic aromatic hydrocarbons (PAHs) adsorbed to the particle surface [20].

Air pollution is also composed of gaseous pollutants including nitrogen dioxide (NO_2_), ozone (O_3_), sulphur dioxide (SO_2_), volatile organic compounds (VOCs), and carbon monoxide (CO). However, these gaseous pollutants except for NO_2_ [21, 22] are not widely reported about their acute or chronic effect relationship with cardiovascular health compared to particulate matters (PM). Ambient NO_2_ is known as a good proxy for traffic-related air pollution alongside the presence of particulate pollutants such as PM_2.5_ and PM_10_ [23]. NO_2_ is part of reactive gas of nitrogen oxides (NO*x*) and produced from combustion processes such as traffic emissions and power plant in the outdoor environment, whereas NO_2_ is generated from unvented heaters and gas stoves in the indoor environment [24]. O_3_ forms from a photochemical reaction between sunlight with the presence of its precursors such as NO_2_ and VOCs which derived it as a secondary gaseous pollutant. Study from ninety largest cities in the United States by the National Morbidity, Mortality, and Air Pollution Study (NMMAPS) observed that cardiopulmonary-related mortality rose by the increment of PM_10_ within a 24-hour period [25]. In another study, daily concentration of PM_10_ was significantly having an impact on ACS incidence, higher on elderly with the highest impact on older men [6]. Moreover, increase in PM_10_ pollution was noted to have relation to rise in frequency of percutaneous coronary interventions (PCIs) in ACS patients [26].

### 1.2. ACS

Cardiovascular disease (CVD) is one of the main causes of mortality and morbidity [27]. One of the important causes of mortality and morbidity related to CVD is acute coronary syndrome (ACS). The estimated incidence of ACS is 141 per 100,000 population per year, and the inpatient mortality rate is approximately 7% [28, 29]. The figures are similar to many developed countries. ACS is a syndrome due to decreased blood flow in the coronary arteries that leads to ischaemia, reduction in heart function, and myocardium cell death [30]. The most common symptom is chest pain, often radiating to the left shoulder or angle of the jaw (crushing, central) and associated with nausea and sweating [31]. ACS is commonly associated with three clinical manifestations, named according to the appearance of the electrocardiogram (ECG): ST elevation myocardial infarction (STEMI, 30%), non-ST elevation myocardial infarction (NSTEMI, 25%), or unstable angina (38%) [32, 33].

ACS occurs due to atherosclerotic plaque (atheroma) rupture, fissure, or ulceration with superimposed thrombosis and coronary vasospasm. Depending on the acuteness, the degree of occlusion, and the presence of collaterals, patients can present as having UA (unstable angina), NSTEMI, or STEMI. In those who have ACS, atheroma rupture is the most found compared to atheroma erosion [34], thus causing the formation of thrombus which blocks the coronary arteries. The diagnosis of ACS requires at least two of the following: ischemic symptoms, diagnostic ECG changes, and serum cardiac marker elevation [35]. ACS often reflects a degree of damage to the coronaries by atherosclerosis. Primary prevention of atherosclerosis is controlling the risk factors: healthy eating, exercise, treatment for hypertension and diabetes, avoiding smoking, and controlling cholesterol levels [36]. The management in ACS includes medication such as antiplatelet, betablocker, ACE inhibitor, lipid lowering agent, anticoagulant, thrombolytic treatment, and invasive percutaneous coronary intervention [37]. Hazard from the exposure to particulate matters has shown to have strong association with ACS that leads to severe detrimental effects on the cardiovascular system through numerous mechanisms including increased inflammation response, oxidative stress, endothelial injury, cell apoptosis, and mitochondrial dysfunction [38]. A study reported that significant 18% increase in the risk of STEMI was linked to each 7.1 g/m^3^ rise in PM_2.5_ concentration before the onset of ACS [39]. MicroRNAs have been shown to contribute as biomarkers in ACS [40]. One study showed, in plasma of myocardial infarction patients, the cardiac myocyte-associated miRNAs, and miR-208b and miR-499 were significantly elevated. Both miRNAs were positively correlated with plasma troponin T, indicating that the release of both was from injured cardiomyocytes [41]. The studies may suggest that miRNAs and PM regulate the cellular pathophysiology of ACS.

### 1.3. miRNAs

miRNAs are estimated to regulate more than 60% of human protein-coding genes at the translational level [42]. These single-stranded nucleotides are the noncoding RNAs, binding to the 3′untranslated region (3′UTR), 5′untranslated region (5′UTR), protein-coding sequence, or gene promoters that lead to degradation or repression of the mRNAs at the posttranscriptional level [43]. miRNAs are involved in various physiological processes including cell cycle, cell proliferation, and apoptosis [43]. Some of the miRNAs are reported in pathological dysregulation including cancer [44], diabetes mellitus [45], and hypertension [46]. In cardiovascular disorder, miRNAs were linked to various pathological events including ACS [40, 47, 48].

In recent years, there is substantial interest on epigenetic regulatory mechanisms at the cellular level and their association with air pollution including miRNAs [49–51]. miRNA, as one of the main epigenetic regulators, has been proposed to modulate the cellular event affected in those who were exposed to PM [7, 8, 52, 53]. The studies suggested that there are changes in miRNA expression in people that are exposed to particulate matter (PM) in air pollution. On top of that, these miRNAs are shown to have association with the genes involved in cardiovascular morbidity [8, 54, 55].

## 2. miRNA Profiles of Air Pollution in ACS

### 2.1. miRNAs and PM

PM_2.5_ exposure from few hours to several weeks can stimulate morbidity and mortality due to cardiovascular disease. In contrast, reductions in PM levels are associated with declines in cardiovascular mortality [56].

A randomised crossover study was designed towards 55 healthy young adults, comparing reduced or ambient levels of indoor PM_2.5_ for 2-week duration; expression of miRNA, mRNA, and protein of 10 serum cytokines was measured. Further analysis showed that higher PM_2.5_ exposure was negatively associated with miR-1-3p, miR-146a-5p, miR-187-3p, miR-199a-5p, and miR-21-5p [7].

In another study, the exposure length was limited to 24 hours. Strong evidence of high expression of let-7d-5p, miR-24-3p, miR-425-5p, miR-4454, miR-4763-3p, miR-502-5p, and miR-505-3p was found after exposure to PM_2.5_ [52].

Turning now to the experimental evidence on myocardial toxicity involvement, Feng et al. reported that PM_2.5_ could contribute to toxicity via miR-205, by negatively regulating the IRAK2/TRAF6/NF-*κ*B signalling pathway [55].

Furthermore, exposure to ambient PM_2.5_ revealed increase level of miR-223-3p expressed in the extracellular vesicle from serum samples [57]. miR-223 was shown to be involved in endovascular inflammation and platelet activation. This serum-derived miRNAs in circulation were identified as cardiovascular mortality predictors in coronary artery disease (CAD) [58].

Li et al. reported that let-7a, miR-146a-5p, and miR-155-5p were highly expressed in respondents exposed to the elevated level of PM exposure and decreased level of interleukin-6 (IL-6) and toll-like receptor 2 (TLR2) mRNAs [9]. However, this study did not specify the type of PM and their specific changes in miRNA and mRNA expression. The experiment utilised benzo[a]pyrene-r-7,t-8,t-9,c-10-tetrahydotetrol-albumin (BPDE-Alb) adducts in serum as the internal exposure biomarker of PM in general.

Similar to the previous experiment, PM_2.5_ exposure upregulated miR-146a, miR-155, and other miRNAs (miR-146b, miR-139, miR-129, miR-340, miR-691, miR-181a, miR-21-3p, and miR-21-5p), and the experiment was set up through intratracheal installation of PM_2.5_ in mice. Furthermore, interleukin-4 (IL-4) was decreased. In contrast, interferon gamma (IFN-*γ*) was increased, and the IL-4/IFN-*γ* ratio was inclined to Th1 shifting. This study concluded that the acute exposure to PM_2.5_ increased the mentioned miRNAs and correlates with T lymphocyte immune imbalance that stimulates Th1-biased immune response [59].

### 2.2. miRNAs and UFP (Ultrafine Particle)

UFP is the ultrasmall and lightweight particle that has been reported to be one of occupational inhalation risks. UFP sizes range from 0.0001 to 0.1 *μ*m [60]. The accumulation of UFP to the lung and various organs could lead to various morbidities including thrombosis, ischaemia, and cardiovascular disease [61, 62]. Acute exposure to UFP in vivo demonstrated increase in inflammation parameters and nitrate stress level, such as serum IL-6, monocyte chemoattractant protein 1 (MCP-1), p47phox (known as NCF1 (neutrophil cytosolic factor 1)), and 3-NT (nitration marker). Furthermore, both of the in vivo and in vitro studies revealed upregulations of miR-301b-3p and let-7c-1-3p with the downstream targets, SMAD2, SMAD3, and transforming growth factor *β*1 (TGF*β*1), indicating higher risk of atherosclerosis following UFP exposure [63]. Another study reported that exposure to UFP among school children was positively associated with increased miR-222 from saliva samples [64]. miR-222 was shown to protect pathological cardiac remodelling and necessary for exercise-induced cardiomyocyte growth and proliferation [65]. It was reported that exposure to UFP for 72 hours significantly downregulates let-7a, let-7b, let-7d, let-7e, miRNA-16, and miRNA-34 with 10-fold upregulation of miR-24 and 6-fold increase in miR-27 and miR-155 expression, respectively [60].

### 2.3. miRNAs and BC (Black Carbon)

The mortality and morbidity related to a BC-related cardiovascular event are reported to be stronger than those due to PM_2.5_ [66]. High concentration of BC was observed to have relation with increased major adverse cardiovascular events in ACS patients [66]. Exposure to BC nanoparticles leads to marked increase in miR-135b and subtle changes in miR-21 and miR-146b, with RT-PCR validation [67]. miR-135b has been shown to stimulate apoptosis and inflammation, reduce cell proliferation, and inhibit macrophage function. Moreover, miR-135b inhibition was reported to increase atherosclerotic plaque development [68]. miR-21 showed increased plaque stability in ACS [69]. This miRNA also showed to have significant elevation in stable and unstable angina and as compared to control subject thus may play an important role for a new biomarker for this disease along with a strong correlation with aging [70]. Exogenous miR-21 was reported to drastically inhibit cardiomyocyte and endothelial cell apoptosis thus leading to significant improvement of cardiac function [71]. The expression of miR-146b was demonstrated to be downregulated in a myocardial infarction model in vivo as compared to the control group, whereas the in vitro experiment showed that the downregulation of miR-146b led to increased inflammatory factors and apoptosis of the vascular cells and was suggested to be associated with the PI3K/Akt/NF-*κ*B pathway [72]. In contrast to earlier findings, however, no evidence of miRNA changes was detected through black carbon exposure through intratracheal instillation in C57BL/6 mice (Julie A [73]).

### 2.4. miRNA and PAHs (Polycyclic Aromatic Hydrocarbons)

miR-155 expression was upregulated in human umbilical cord vein cells (HUVECs) exposed to PAH treatment. The putative gene target for miR-155 was shown to be linked to Wingless/Integrated (Wnt) and epidermal growth factor (ErbB) signalling which is important for vasculature development, thus proposing involvement of miRNA for a novel target for cardiovascular-related therapy [8]. In one study, exposure to PAHs showed significant increase in lower expression of miR-24-3p, miR-27a-3p, miR-142-5p, and miR-28-5p, through analysis of urinary 4-hydroxyphenanthrene and/or plasma BPDE-Alb. On the other hand, urinary 1-hydroxynaphthalene, 2-hydroxynaphthalene, 2-hydroxyphenanthrene, and the sum of monohydroxy-PAHs were associated with high level of miR-150-5p expression [74]. In PAH-induced hepatocarcinogenesis, miR-181a, miR-181b, and miR-181d were significantly upregulated through p38 MAPK activation [75]. The summary of specific particulate matter and related miRNAs is shown in Table 2.

## 3. PM Exposure and Cardiac Inflammatory Mediators

PM exposure is known to exacerbate inflammatory response through mRNA mediation [63, 76, 77], some of it through miRNA modulation [7–9]. The summary of particulate matter (PM) and related cardiac inflammatory mediators is shown in Table 3. In one experiment, exposure to daily PM_10_ of overweight and obese patients showed an inverse association of DNA methylation of inflammatory genes, in particular cluster of differentiation antigen 14 (CD14) and toll-like receptor 4 (TLR4), but not in tumour necrosis factor-*α* (TNF-*α*) [78]. Through in vitro study, PM_2.5_ was shown to decrease cell viability and increased the expression of NF-kB1 family gene mRNA and inflammatory mediators including C-reactive protein (CRP), cyclooxygenase-2 (COX-_2_), interleukin-1*β* (IL-1*β*), interleukin-8 (IL-8), interleukin-12 (IL-12), and TNF-*α* (J. [79]). In a meta-analysis finding, short-term exposure to PM_2.5_ and PM_10_ had a significant association with inflammation and blood coagulation markers, TNF-*α*, and fibrinogen; however, the long-term exposure to both PM_2.5_ and PM_10_ was not significant [80]. In another study, it was reported that the smaller the size of the PM, the more significant the association of PM exposure to inflammation markers, for example, IL-1*β*, IL-6, and TNF-*α* [81]. Exposure to PM_2.5_ was also associated with increased level of proinflammatory cytokines MCP-1, macrophage inflammatory protein 1*α*/*β* (MIP-1*α*/*β*), IL-6, and IL-1*β* and increased level of inflammatory response, for example, CD4+, CD8+, CD14+, and CD16+ [82]. MCP-1 acts through the CCR2 receptor and is increased by chronic inflammatory condition which stimulates the adherence of monocytes to the subendothelial region of the atherogenic arterial wall [83]. This finding is supported by a Mendelian randomisation study, indicating the linkage of MCP-1 to an increased risk of coronary artery disease (CAD), myocardial infarction (MI), and ischemic stroke [84]. In several atherosclerotic studies, the inhibition of the CCR2 receptor reduced inflammatory monocyte recruitment, reduced neointimal hyperplasia, and reduced the size of atherosclerotic plaque [85–87]. One study among healthy nonsmokers exposed to ambient PM_2.5_ for 24 hours demonstrated significant increase in proinflammatory cytokines (IL-6 and IL-1*β*) and antiangiogenic agent (TNF-*α*) that may contribute to endothelial dysfunction, inflammation, and platelet activation. PM_2.5_ induced reactive oxygen species (ROS), increased cardiomyocyte apoptosis, stimulated inflammatory cell infiltration, and enhanced the inflammatory factors in AC16 cells and heart tissue [55]. The regulation is through PM_2.5_-induced downregulation of miR-205 activating the TNF receptor-associated factor 6 (TRAF6)/nuclear transcription factor-B (NF-*κ*B) signalling pathway, which further activated the signalling network. In the higher PM2.5 exposure, the result showed a positive association with IL-1, IL-6, and TNF [7].

Exposure to UFPs was reported to have a significant link to cardiovascular disease, such as atherosclerosis (AS) [63]. Mice were given an acute dose of UFPs for six days, after which they were sacrificed few days later. Postacute exposure revealed increased inflammation responses and nitrate stress, with elevated IL-6, MCP-1, p47phox, and 3-NT levels in the mouse serum compared to the untreated control. In an in vitro study, exposure to diesel UFP increased the levels of IL-6 and vascular endothelial growth factor (VEGF) in lung epithelial cells, whereas treatment of endothelial cells with diesel UFP media increased the levels of vascular cell adhesion protein 1 (VCAM-1) and intercellular adhesion molecule 1 (ICAM-1) in endothelial cells [88].

Black carbon (BC) is a traffic-related particle that is formed as a combustion by-product and has been linked to several cardiovascular adverse events, stronger than the effect of fine PM_2.5_ [89]. However, this study contrasts with that of Bourdon et al.'s finding few years before who stated that intrathecal instillation of carbon black nanoparticles had no effect on cardiac gene expression [73]. This finding was supported by Dominguez-Rodriguez et al. who argue that the major adverse cardiovascular events (MACE) were detected in other PM exposure, except BC [90]. Nevertheless, BC exposure was linked to methylation of genes implicated in inflammation and endothelial function, through decreased coagulation factor III (*F3*) and ICAM-1 methylation [76].

1-Hydroxypyrene (1-OHP) is a biomarker for traffic-related air pollution exposure to polycyclic aromatic hydrocarbons (PAHs). In one study, urinary 1-OHP levels were higher in taxi drivers than in nonoccupationally exposed people, and it was linked to proinflammatory cytokines, for example, IL-1*β*, IL-6, IL-10, TNF-*α*, IFN-*γ*, and hs-CRP [77]. These higher inflammatory biomarkers were linked to the key indicators of cardiovascular morbidity.

## 4. miRNAs and PM-Induced Inflammation Mediators

Even though study on PM-induced cardiac inflammation mediators is limited, other experiments were performed utilising the similar genes or mediators in other system rather than focusing on the cardiovascular system. Regardless, these mediators are similar and involved in many parts of the ACS cellular pathological mechanism. For example, CD14 gene silencing was reported to significantly change the expression of 199a-3p, miR-199a-5p, and miR-21-5p in the RAW264.7 macrophage cell line [91]. Endothelin-1 (ET-1) is a peptide encoded by the ET-1 gene (EDN1). Endothelin-1 (ET-1) is a 21-amino acid polypeptide that is mainly generated by endothelial cells of the vascular system and has been shown to participate in a variety of biological processes, including inflammation, fibrosis, proliferation of vascular smooth muscle, and cardiovascular hypertrophy [92]. ET-1 is a potent vasoconstrictor in cardiac, renal, and nervous system vasculature and known to be attenuated by miRNA regulation [93]. In one study, overexpression of miR-199 inhibited ethanol-induced EDN1, and on the other hand, inhibiting miR-199 levels led to increase in ET-1 protein with the presence of ethanol [94]. IL-1 is a cumulative IL-1*α* and IL-1*β* and a potent inducer of inflammatory protein, and miR-448 reportedly enhanced the production of proinflammatory cytokine including IL-1*β* [95]. IL-6 is a proapoptotic and proinflammatory cytokine, secreted by inflammatory cells in response to inflammation. In IL-6-treated cells, miR-181c was significantly downregulated as compared to control cells [96]. Other study found that miR-410 targets the 3′untranslated region (3′UTR) of IL-6; thus, increase in miR-410 reduces the IL-6 levels [97]. miR-124a is reported to bind at the 3′UTR of MCP-1 mRNA, and the upregulation of this miRNA supressed the MCP-1 protein levels [98]. Toll-like receptor 2 (TLR2) plays an important role in the activation of innate immunity, activation of the macrophage, and promotion of apoptosis. Some study reported that miR-146a [99] and miR-105 [100] downregulated the expression of TLR2. On the other hand, miR19a/b upregulated the TLR2 expression, and the increased expression of miR-19a/b by mimics significantly reduced TLR2 protein and inhibited the TLR2-triggered cytokine and kinase activities [101]. TNF-*α* is a proinflammatory cytokine. Luciferase reporters confirm the in silico algorithms of miR-181a-5p target 3′UTR of TNF-*α*, in which the miR-181a-5p mimic suppressed the TNF-*α* levels and the miR-181a-5p inhibitor increased its level [102]. The summary of inflammatory genes and related miRNAs is shown in Table 4.

## 5. Discussion and Conclusion

**Figure 1:**
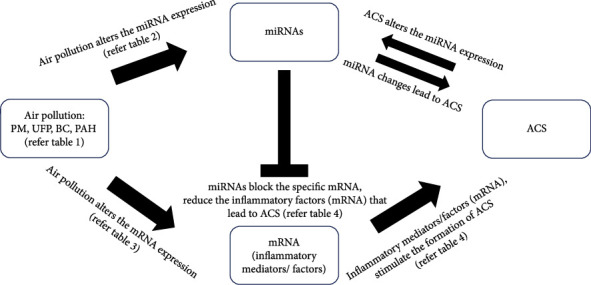
The summary of the mode of action of PMIA through miRNA-mRNA regulation. ACS = acute coronary syndrome; BC = black carbon; miRNA = microRNA; mRNA = messenger RNA; PAH = polycyclic aromatic hydrocarbon; PM = particulate matter; PMIA = particulate matter-induced ACS; UFP = ultrafine particle.

**Figure 2:**
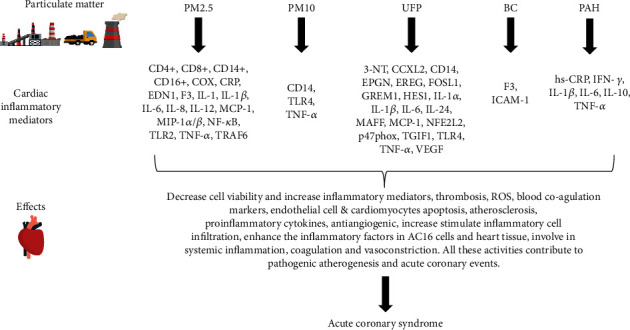
The mode of action of PM in inducing ACS. The PM stimulates the production cardiac inflammatory mediators that might lead to ACS. 3-NT = nitration marker; ACS = acute coronary syndrome; CCXL2 = chemokine genes; CD14 = differentiation antigen 14; CD14+ = cluster of differentiation 14 (monocyte differentiation antigen); CD16+ = cluster of differentiation 16 (monocyte differentiation antigen); CD4+ = cluster of differentiation 4 (monocyte differentiation antigen); CD8+ = cluster of differentiation 8 (monocyte differentiation antigen); COX = cyclooxygenase; CRP = C-reactive protein; EDN1 = endothelin 1 (protein-coding gene); EPGN = epithelial mitogen (protein-coding gene); EREG = epiregulin (protein-coding gene); F3 = coagulation factor III, tissue factor (protein-coding gene); FOSL1 = FOS like 1, AP-1 transcription factor subunit (protein-coding gene); GREM1 = gremlin 1, DAN family BMP antagonist (protein-coding gene); HES1 = Hes family BHLH transcription factor 1 (protein-coding gene); hs-CRP = high-sensitivity C-reactive protein; ICAM-1 = intercellular adhesion molecule 1; IFN-*γ* = interferon gamma; IL-1 = interleukin-1; IL-10 = interleukin-10; IL-12 = interleukin-12; IL-1*α* = interleukin-1*α*; IL-1*β* = interleukin-1*β*; IL-24 = interleukin-24; IL-6 = interleukin-6; IL-8 = interleukin-8; MAFF = MAF BZIP transcription factor F (protein-coding gene); MCP-1 = monocyte chemoattractant protein 1; MIP-1*α*/*β* = macrophage inflammatory protein-1 alpha/beta; NFE2L2 = nuclear factor erythroid 2-like 2 (protein-coding gene); NF-*κ*B = nuclear factor kappa B; p47phox = neutrophil cytosolic factor 1 protein, encoded by NCF1; PM = particulate matter; TGIF1 = TG-interacting factor 1; TLR2 = toll-like receptor 2; TLR4 = toll-like receptor 4; TLR4 = toll-like receptor 4; TNF-*α* = tumour necrosis factor-*α*; TRAF6 = TNF receptor-associated factor 6; VEGF = vascular endothelial growth factor.

miRNAs bind to a specific sequence in the 3′UTR, 5′UTR, and coding sequence of their target mRNAs, resulting in translational repression, mRNA deadenylation, and decapping of the target mRNAs, respectively [103]. Therefore, we may take various viewpoints on PM exposure to miRNA and mRNA involved in inflammation and ACS (Figure 1). Firstly, this paper might suggest that PM could deter the level of mRNA involved in cardiac inflammation (Figure 2). For example, PM_2.5_ was reported to increase TNF-*α*, CD14+, and TLR2. These mRNAs and others (Table 3) might have a significant potential role for future applications in ACS screening and biomarkers. Secondly, we would suggest specific miRNA that could be used as therapeutic tools to treat ACS. We identify several studies indicating significant miRNA functions to downregulate several inflammatory factors, such as miR-146a-5p, miR-181a, miR-199a-5p, and miR-21-5p, which target TRL2, TNF-*α*, and CD14, respectively (Table 4). An increase in the stated miRNAs could downregulate the genes involved in cardiac inflammation and grant future experiments and validation. Therefore, a common pathway in PMIA regulated by the miRNAs could be investigated in the future.

The findings presented here have established that PMIA has a major impact on miRNAs and vice versa. For prevention and management of ACS, PM and other hazardous substances from air pollution should be considered significant modifiable risk factors. Policymakers should strengthen the effort to reduce air pollution exposure through suitable and effective legislation significantly. The use of miRNA and mRNA in novel clinical settings offers promising potentials. Currently, multiple clinical trials are exploring miRNA profiles in various illness conditions for prognostic or diagnostic reasons. Since one miRNA can target several different mRNAs, it is important to be cautious when attributing miRNA effects to a specific mRNA. miRNAs have been well established to play a role in the regulation of inflammatory factors in ACS. However, little is known on how these miRNAs directly contribute to the modulation of PMIA. Further study should be conducted to determine the best techniques for air pollution reduction and to document the effects of these techniques on the incidence of ACS and its relationship to morbidity and mortality. It seems prudent to predict that future research investigating PMIA on miRNAs will adopt more comprehensive technology.

## Figures and Tables

**Table 1:** Summary of particulate matter (PM) and its composition sources.

Particulate matter (PM)	Main source of emission	Reference
PM_2.5_	Traffic emissions (brake and vehicle exhaust) Industrial emissions released from power plant and oil refinery Secondary organic and inorganic aerosol	[11] ([104]; [105]; [14])
PM_10_	Road dust and tire wear Construction activities Wildfires and windblown dust	([106]; [107])
Ultrafine particles (UFP)	Tailpipe emissions from vehicle exhaust, diesel combustion, and solid biomass burning	([106]; [11]; [18]; [108]; [88])
Black carbon (BC)	Combustion process (vehicle exhaust emissions and cookstoves)	([109]; [106])
Polycyclic aromatic hydrocarbons (PAHs)	Incomplete combustion of fossil fuels, natural combustion (forest fires and volcanic eruption), anthropogenic cause (wood, coal burning, vehicle exhaust emissions, heat and power generation)	([110]; [16]; [111]; [17])

**Table 2:** Summary of particulate matter (PM) and related miRNAs.

Particulate matter (PM)	miRNAs	Reference
PM_2.5_	miR-129, miR-139, miR-146a, miR-146b, miR-155, miR-340, miR-691, miR-181a, miR-21-3p, and miR-21-5p	[59]
	miR-1-3p, miR-146a-5p, miR-187-3p, miR-199a-5p, and miR-21-5p	[7]
	let-7d-5p, miR-24-3p, miR-425-5p, miR-4454, miR-4763-3p, miR-502-5p, and miR-505-3p	[52]
	miR-205	[55]
	miR-223-3p	[57]

PM (general)	let-7a, miR-146a-5p, and miR-155-5p	[9]

Ultrafine particle (UFP)	miR-222	[64]
	let-7a, let-7b, let-7d, let-7e, miRNA-16, miR-155, miR-24, miR-27, and miRNA-34	[60]
	miR-301b-3p and let-7c-1-3p	[63]

Black carbon (BC)	miR-135b, miR-146b, and miR-21	[67]

Polycyclic aromatic hydrocarbons (PAHs)	miR-181a, miR-181b, and miR-181d	[75]
	miR-24-3p, miR-27a-3p, miR-142-5p, and miR-28-5p and miR-150-5p	[74]
	miR-155	[8]

**Table 3:** Summary of particulate matter (PM) and related cardiac inflammatory mediators.

Particulate matter (PM)	Cardiac inflammatory mediators	Effect	Reference
PM_2.5_	IL-1*β*, IL-8, IL-12, TNF-*α*, CRP, COX	Decrease cell viability and increase inflammatory mediators	[79]
	TNF-*α*	Increase inflammation and blood coagulation markers	[80]
	CD4+, CD8+, CD14+ and CD16+, IL-6, IL-1*β*, MCP-1, MIP-1*α*/*β*, TNF-*α*	Increased endothelial cell apoptosis, proinflammatory cytokines, and antiangiogenic activity contribute to pathogenic atherogenesis and acute coronary events	[82]
	TRAF6, NF-*κ*B	Increased reactive oxygen species (ROS), increased cardiomyocyte apoptosis, stimulated inflammatory cell infiltration, and enhanced inflammatory factors in AC16 cells and heart tissue	[55]
	EDN1, F3, IL-1, IL-6, TNF, TLR2	Involved in systemic inflammation, coagulation, and vasoconstriction	[7]
Smaller PM size	IL-1*β*, IL-6, TNF-*α*	Increase inflammation marker	[81]
PM_10_	CD14 and TLR4	Inverse association of DNA methylation of inflammatory genes in overweight and obese patients	[78]
	TNF-*α*	Increase inflammation and blood coagulation markers	[80]
Ultrafine particle	3-NT, CCXL2, EPGN, EREG, FOSL1, GREM1, HES1, IL-1*α*, IL-1*β*, IL-6, IL-24, NFE2L2, MAFF, MCP-1, p47phox, TGIF1, VEGF	Biological dysregulation in atherosclerosis, increase inflammation	[19]; [63])
Black carbon (BC)	F3, ICAM-1	Inflammation and thrombosis	[76]
Polycyclic aromatic hydrocarbons (PAHs)	IL-1*β*, IL-6, IL-10, TNF-*α*, IFN-*γ*, and hs-CRP	Inflammation and atherogenesis	[77]

**Table 4:** Target inflammatory genes and miRNAs.

Target inflammatory genes	miRNAs	Related cell/organ/disease	Author
CD14	miR-199a-3p, miR-199a-5p, and miR-21-5p	RAW264.7 macrophage cell line, lipopolysaccharide- (LPS-) induced proinflammatory cytokine release, and LPS-induced septic shock	[91]
EDN1 (putative target of miRNA	miR-199	Liver sinusoidal endothelial cells (rLSEC) derived from ethanol-fed rats	[94]
IL-1*β*	miR-448	Autoimmune diseases	[95]
IL-6	miR-181c	INS-1 cells	[96]
IL-6	miR-410	Lupus nephritis, systemic lupus erythematosus (SLE) in kidney tissue of MRL/lpr mice	[97]
MCP-1	miR-124a	Synoviocytes from rheumatoid arthritis	[98]
TLR2	miR-146a	BLP-stimulated human THP-1 promonocytic cells, innate immune response to infection	[99]
TLR2	miR-105	Primary human keratinocytes, challenge with *Porphyromonas gingivalis* (a Gram-negative bacterium that triggers TLR-2 and TLR-4)	[100]
TLR2	miR19a/b	Rheumatoid fibroblast-like synoviocytes, rheumatoid arthritis (RA)	[101]
TNF-*α*	miR-181a-5p	Dendritic cells	[102]

## Abbreviations

1-OHP: 1-Hydroxypyrene
3′UTR: 3′untranslated region
3-NT: Nitration marker
5′UTR: 5′untranslated region
ACS: Acute coronary syndrome
AD: Aerodynamic diameter
Akt: Protein kinase B (PKB)
BC: Black carbon
BPDE-Alb: Benzo[a]pyrene-r-7,t-8,t-9,c-10-tetrahydotetrol-albumin
CAD: Coronary artery disease
CCR2: C-C chemokine receptor type 2
CCXL2: Chemokine genes
CD14: Differentiation antigen 14
CD14+: Cluster of differentiation 14 (monocyte differentiation antigen)
CD16+: Cluster of differentiation 16 (monocyte differentiation antigen)
CD4+: Cluster of differentiation 4 (monocyte differentiation antigen)
CD8+: Cluster of differentiation 8 (monocyte differentiation antigen)
CO: Carbon monoxide
COX: Cyclooxygenase
COX-2: Cyclooxygenase-2
CRP: C-reactive protein
CVD: Cardiovascular disease
CYP: Cytochrome P450
DNA: Deoxyribonucleic acid
ECG: Electrocardiogram
EDN1: Endothelin 1 (protein-coding gene)
EPGN: Epithelial mitogen (protein-coding gene)
ErbB: Epidermal growth factor (EGF)
EREG: Epiregulin (protein-coding gene)
ET-1: Endothelin 1
F3: Coagulation factor III, tissue factor (protein-coding gene)
FOSL1: FOS kike 1, AP-1 transcription factor subunit (protein-coding gene)
GREM1: Gremlin 1, DAN family BMP antagonist (protein-coding gene)
HES1: Hes family BHLH transcription factor 1 (protein-coding gene)
hs-CRP: High-sensitivity C-reactive protein
HUVECs: Human umbilical cord vein cells
ICAM-1: Intercellular adhesion molecule 1
IFN-*γ*: Interferon gamma
IL-1: Interleukin-1
IL-12: Interleukin-12
IL-1*α*: Interleukin-1*α*
IL-1*β*: Interleukin-1*β*
IL-24: Interleukin-24
IL-4: Interleukin-4
IL-6: Interleukin-6
IL-8: Interleukin-8
IL-10: Interleukin-10
IRAK2: Interleukin-1 receptor-associated kinases
MACE: Major adverse cardiovascular events
MAFF: MAF BZIP transcription factor F (protein-coding gene)
MAPK: Mitogen-activated protein kinase
MCP-1: Monocyte chemoattractant protein 1
MI: Myocardial infarction
MIP-1*α*: Macrophage inflammatory protein-1 alpha
MIP-1*β*: Macrophage inflammatory protein-1 beta
miRNAs: MicroRNAs
mRNA: Messenger RNA
NFE2L2: Nuclear factor erythroid 2-like 2 (protein-coding gene)
NF-*κ*B: Nuclear factor kappa B
NMMAPS: National Morbidity, Mortality, and Air Pollution Study
NO_2_: Nitrogen dioxide
NO*x*: Nitrogen oxides
NSTEMI: Non-ST elevation myocardial infarction
O_3_: Ozone
p47phox: Neutrophil cytosolic factor 1 (NCF1)
PAHs: Polycyclic aromatic hydrocarbons
PI3K: Phosphatidyl-inositol-3-kinase
PM: Particulate matter
PMIA: Particulate matter-induced ACS
RNA: Ribonucleic acid
ROS: Reactive oxygen species
RT-PCR: Real-time polymerase chain reaction
SDG: Sustainable Development Goals
SMAD2: SMAD family member 2
SMAD3: SMAD family member 3
SO_2_: Sulphur dioxide
STEMI: ST elevation myocardial infarction
TGF*β*1: Transforming growth factor *β*1
TGIF1: TG-interacting factor 1
Th1: T helper type 1
TLR2: Toll-like receptor 2
TLR4: Toll-like receptor 4
TNF-*α*: Tumour necrosis factor-*α*
TRAF6: TNF receptor-associated factor 6
UA: Unstable angina
UFP: Ultrafine particle
UN: United Nation
VCAM-1: Vascular cell adhesion protein 1
VEGF: Vascular endothelial growth factor
VOCs: Volatile organic compounds
WHO: World Health Organization
Wnt: Wingless/Integrated.
